# Value of total cholesterol readings earlier versus later in life to predict cardiovascular risk

**DOI:** 10.1016/j.ebiom.2021.103371

**Published:** 2021-05-14

**Authors:** Andreas Leiherer, Hanno Ulmer, Axel Muendlein, Christoph H. Saely, Alexander Vonbank, Peter Fraunberger, Bernhard Foeger, Eva Maria Brandtner, Wolfgang Brozek, Gabriele Nagel, Emanuel Zitt, Heinz Drexel, Hans Concin

**Affiliations:** a Vorarlberg Institute for Vascular Investigation and Treatment (VIVIT), Carinagasse 47, Feldkirch A-6800, Austria; b Agency for Preventive and Social Medicine, Bregenz, Austria; c Department of Medical Statistics, Informatics and Health Economics, Innsbruck Medical University, Innsbruck, Austria; d Department of Internal Medicine I, Academic Teaching Hospital Feldkirch, Feldkirch, Austria; e Private University of the Principality of Liechtenstein, Triesen, Liechtenstein; f Institute of Epidemiology and Medical Biometry, Ulm University, Ulm, Germany; g Medical Central Laboratories, Feldkirch, Austria; h Department of Internal Medicine III, Academic Teaching Hospital Feldkirch, Feldkirch, Austria; i Drexel University College of Medicine, Philadelphia, PA, United States; j Department of Internal Medicine, Academic Teaching Hospital Bregenz, Bregenz, Austria

**Keywords:** Cholesterol, Aging, Advanced age, Cardiovascular risk factors, SCORE, Risk prediction, AUC, area under the curve, CAD, coronary artery disease, CVOS, cardiovascular observation study, EAS, European Atherosclerosis Society, eGFR, estimated glomerular filtration rate, ESC, European Society of Cardiology, HS, health survey, ROC, receiver operating characteristics, SCORE, Systematic COronary Risk Estimation, TC, total cholesterol, TC_CVOS_, total cholesterol at cardiovascular observation study, TC_HS_, total cholesterol at health survey

## Abstract

**Background:**

Prognostic implications of blood cholesterol may differ at different stages of life. This cohort study compares the value of total cholesterol (TC) readings earlier versus later in life for the prediction of coronary atherosclerosis, cardiovascular events, and cardiovascular death.

**Methods:**

In a cardiovascular observation study (CVOS) we performed coronary angiography and prospectively recorded cardiovascular events in 1090 patients over up to 19 years. These patients had participated in a health survey (HS) 15 years prior to the CVOS baseline. TC was measured twice, first at the earlier HS and then later at CVOS recruiting.

**Findings:**

Patients in the highest versus the lowest TC-category of the HS had an OR of 4.30 [2.41–7.65] for significant CAD at angiography, a HR of 1.74 [1.10–2.76] for cardiovascular events, and a HR of 7.55 [1.05–54.49] for cardiovascular death after multivariate adjustment. In contrast, TC as measured at the baseline of the CVOS was neither significantly associated with significant CAD (OR= 0.75 [0.49–1.13]) nor with cardiovascular events or death during follow-up (HR= 0.86 [0.62–1.18] and 0.79 [0.41–1.53], respectively). Moreover, the ESC/EAS-SCORE was found to be more powerful in predicting cardiovascular mortality when using earlier instead of later TC, with a continuous net reclassification improvement of 0.301 (*p*<0.001).

**Interpretation:**

Early measurement not only enables early intervention in keeping with the concept of lifelong exposure to atherogenic lipoproteins. These data also suggest that cardiovascular risk prediction is more accurate if using earlier in life TC readings.

**Funding:**

The present study did not receive any particular funding

**Research in context**

**Evidence before this study**

At an advanced age, cardiovascular risk prediction becomes a bigger issue for most people than it was earlier when they were young or in their midlife. Total cholesterol (TC) has always been one of the most commonly used parameters for risk prediction and it is also part of the prominent SCORE prediction chart for cardiovascular risk in the recent ESC/EAS guidelines in Europe. However, it is not clear, what kind of TC reading data are more valuable for cardiovascular risk prediction of elderly patients: Current readings or past readings taken some 15 years ago?

**Added value of this study**

In the present study, we compared TC readings from a large Austrian health survey (HS) of clinically healthy and statin-naive individuals in the 1980ies to TC readings of the identical subjects 15 years later, who by then had become cardiovascular risk patients. Performing coronary angiography and recording patients` fatal and non-fatal cardiovascular events for another 19 years, we found (i) that TC readings, when assessed earlier in life, were a better predictor of coronary atherosclerosis, cardiovascular events, and cardiovascular mortality than the actual lipid profile of elderly people and (ii) that TC readings earlier in life significantly improved the accuracy of cardiovascular risk prediction over and above readings later in life, even comprising LDL-C and HDL-C.

**Implications of all the available evidence**

TC readings earlier in life, when people are healthy and untreated, are of high prognostic value and allow improved later-life risk prediction.

Alt-text: Unlabelled box

## 1 Introduction

Ever since the early reports from the Framingham study [1], total serum cholesterol (TC) has become a standard in risk factor evaluation in human epidemiology and clinical medicine and as such is embedded in the Systematic COronary Risk Estimation (SCORE) chart predicting the risk for fatal cardiovascular disease in European populations [2]. According to SCORE, the 10-year risk for fatal cardiovascular events increases by approximately a factor of 4 between the ages of 50 and 65, provided that the other risk factors including TC remain constant [3]. This quadrupling of risk within 15 years raises the question whether TC measured in the past, e.g. at the age of 50, has superior diagnostic value over more recent measurements, e.g. taken at the age of 65.

Of interest is the notion that low density lipoprotein-cholesterol (LDL-C) concentration, although within the normal range, is associated with atherosclerosis [4]. On the other hand, patients hospitalized with coronary artery disease (CAD) had lower LDL-C than the general population and nearly every other patient had an LDL-*C* <100 mg/dL (2.6 mmol/L) [5]. Moreover, data from the MIRACL trial demonstrate that, in patients with acute coronary syndrome, neither LDL-C nor TC were predictive for future cardiovascular events [6]. Given the fact that acute disease states, medication, comorbidities, advanced age, or frailty may confound cholesterol measurements later in life, a past measurement may be of value. This opens the question to what extent TC is a good predictor of disease and at which stage of life, mid adulthood versus late adulthood. Taking into account the long asymptomatic latent period of atherosclerosis, this question is closely related to the one whether TC is a predictor of cardiovascular events for the clinically healthy individual as well as for the patient with established cardiovascular disease.

We had the unique opportunity to combine data sets of one population recruited by two studies being 15 years apart. The first came from a large health survey (HS) of clinically healthy individuals [7,8] and the second from a recent prospective cardiovascular observation study (CVOS) initiated 15 years later on the same patients undergoing coronary angiography. Hence, the aim of the present study was to investigate whether TC in the healthy and untreated state or TC at coronary angiography 15 years later is the better predictor of the cardiovascular risk in elderly patients.

## 2 Methods

### 2.1 Study subjects

**Fig. 1:**
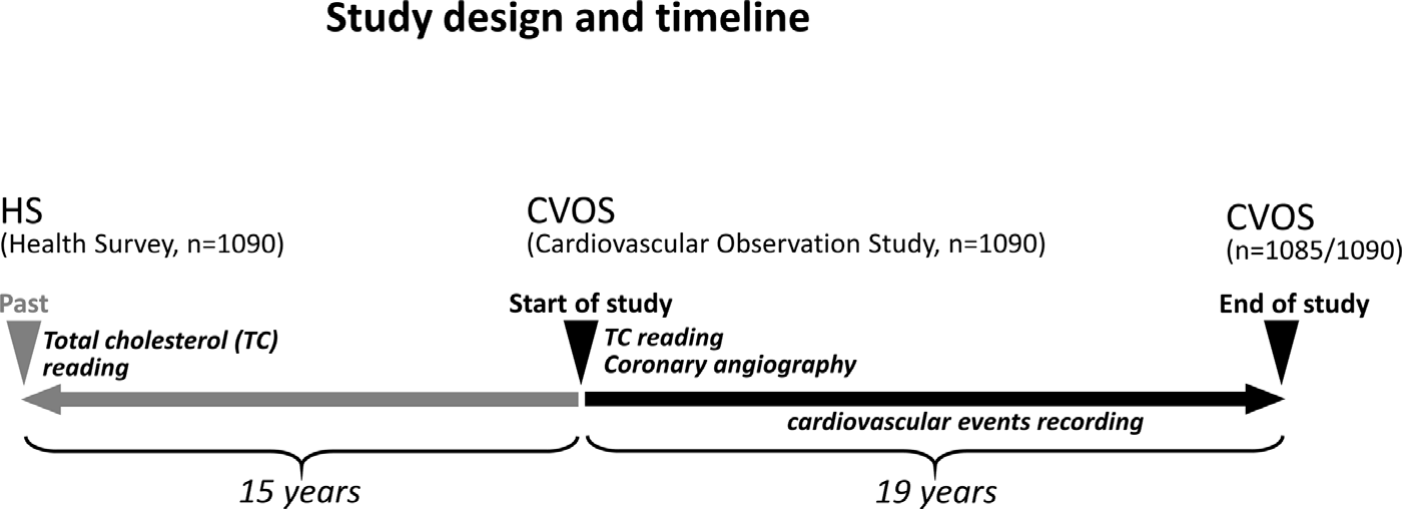
Study design and timeline.

This study (**supplementary figure 1**) comprises 1090 participants of Caucasian origin with a median age of 65 years living in Vorarlberg, the westernmost province of Austria in Central Europe, who participated in a cardiovascular observation study (CVOS), as is shown in Fig. 1. These patients were cardiovascular risk patients, all undergoing coronary angiography for the evaluation of established or suspected stable CAD. Recruitement started in 1999 and patients were follow for up to 19 years. A follow up examination took place every two years.

A median period of 15 years prior to that, all of the 1090 cardiovascular risk patients included here had participated in a large HS, the Vorarlberg Health Monitoring & Prevention Program [7,8], that comprised over 185,000 adult residents of Vorarlberg, which accounted for 50% of the population of Vorarlberg at the time. HS enrolment was voluntary and costs were covered by the participants' health insurance. All subjects participated solely in the context of medical prevention and did not see their physician for any signs or symptoms of cardiovascular or other disease. At the time of HS recruiting, all subjects were statin-naïve, whereas at the CVOS baseline, 46% were taking statins. Statin doses and their equivalents were standardized as described previously [9] according to the LDL lowering potency as defined by the U.S. federal drug administration [10].

This study thus contains (i) laboratory data from 1090 subjects in a healthy condition, (ii) laboratory data from the same subjects, when they were 15 years older, by then suspected to have CAD, and thus referred for coronary angiography, and (iii) follow-up data for another 19 years, altogether covering a time-span of up to 33 years.

### 2.2 Clinical and laboratory analyses and study endpoints

Basic clinical data were assessed as described in detail previously [8,11]. Laboratory analyses, in particular the measurement of TC in single human samples were done at the medical central laboratories Feldkirch and Dornbirn according to consistent protocols at HS and later at CVOS [8,11]. TC concentrations were stratified into four categories (≤4.4, 4.5–5.4, 5.5–6.4, and ≥6.5 mmol/L) resulting in median concentrations of 4.2, 5.1, 6.0, and 7.2 mmol/L respectively, which reflects the categorization of TC concentrations used in the SCORE charts of the current 2019 ESC/EAS guidelines (4, 5, 6, and 7 mmol/L) [12]. Apart from TC, systolic blood pressure, age, gender, and the status of current smoking are used for cardiovascular risk prediction in the SCORE chart [12]. According to the chart for low risk European countries, with Austria being one of them [12], four risk categories of the 10-year risk of cardiovascular death were specified (green category, low risk of <3%; yellow category, medium risk of 3–4%; red category, high risk of 5–9%; and dark red category, very high risk of ≥10%) [12].

Type 2 diabetes mellitus (T2DM) was diagnosed according to the World Health Organization guidelines [13]. Body mass index (BMI) was calculated as body weight (kg)/height^2^ (m).

Coronary angiography was performed in all 1090 patients referred solely for clinical reasons by their physicians. All visible lesions were recorded. Significant CAD was diagnosed in the presence of any significant coronary artery stenoses with lumen narrowing ≥50%. The extent of CAD was defined as the number of significant coronary stenoses, as described previously [14], and the severity of CAD according to the number of diseased vessels (one-, two-, and three-vessel disease).

Prospectively, the 1090 patients were followed for up to 19 years (median 11.2 years, interquartile range (IQR)= 9.1–12.7 years). Complete follow-up data were available for 1085 out of 1090 patients, amounting to a follow-up rate of >99%. The primary study endpoint was cardiovascular death (fatal myocardial infarction, sudden cardiac death, mortality from congestive heart failure due to CAD) and the secondary endpoint a composite of cardiovascular death, fatal ischemic stroke, non-fatal myocardial infarction, non-fatal ischemic stroke and need for coronary artery bypass grafting, percutaneous coronary intervention, or revascularization in the carotid or peripheral arterial beds.

### 2.3 Ethics statement

The present study conforms to the ethical guidelines of the 1975 Declaration of Helsinki and has been approved by the Ethics Committee of Vorarlberg, Austria, and the University of Innsbruck, Austria (EK-2-22013/0008 and EK-Nr. 2006-6/2). All participants gave written informed consent.

### 2.4 Statistical analysis

Normal distribution was checked using the Kolmogorov–Smirnov test. Non-normally distributed variables were log10 transformed. For standardization all continuous variables were z-transformed when comparing scores from disparate distributions. Differences between baseline characteristics of the HS and the CVOS were tested for statistical significance with the paired McNemar test for categorical and Wilcoxon test for continuous variables. Correlation analyses were performed calculating non-parametric Spearman rank correlation coefficients (Spearman test). For prediction of significant CAD at angiography, logistic regression analysis was used and odds ratios (OR) were given together with the 95% confidence intervals in square brackets and the respective p-value. For estimating the extent of CAD, analysis of covariance (ANCOVA) models were built using a general linear model approach for calculating the respective F-value (F) and p-value. A linear regression model was used to check for collinearity among variables. For prognostic endpoints, adjusted hazard ratios (HRs) for the incidence of vascular events during 19 years of follow up were derived from Cox proportional hazards models test (Cox regression). Similar to ORs, HRs were given together with the 95% confidence intervals in square brackets and the respective p-value. The proportional hazard assumption was checked by examination of scaled Schoenfeld residuals. Covariates that were adjusted for in the regression and Cox models were age, time between HS and CVOS (∆age), gender, BMI, systolic blood pressure, estimated glomerular filtration rate (eGFR), current smoking status, and T2DM status.

All missing values were missing completely at random (MCAR) according to Little's MCAR test [15]. For regression analysis with respect to cardiovascular outcomes we used multiple imputation for missing values of parameter BMI_CVOS_ (*n* = 4) imputing median values of 5 imputation estimates applying Markov Chain Monte Carlo method with Predictive Mean Matching. All other parameters had no missing values or were analyzed according to complete case analysis.

To examine the potential utility of predictive biomarkers [16], composed models were compared according to their time-independent receiver operating characteristic (ROC] curves applying DeLong's test [17] or by calculating Harrell´s C and Somers’ D for time-dependent ROC curves [18,19]. The corresponding area under the curve (AUC) was calculated using the pROC and survivalROC package for R as described elsewhere [20,21]. The integrated discrimination improvement (IDI) index and the continuous net reclassification improvement (cNRI) index were calculated for mean follow-up time using the survIDINRI package [22,23].

Moderation analysis was performed running the PROCESS procedure for SPSS version 3.5 [24]. A priori power calculation showed that, in the event that the standard deviation is half of the population mean and given that the test and control group contain 615 and 475 patients, respectively (as it is the case for significant CAD), the power of the study to detect a between group difference of only 10% would be 91%. (SPSS Sample Power 3.0, SPSS, Inc., Chicago, IL). All statistical analyses were performed with SPSS 25.0 for Windows (IBM corp., USA), and R statistical software v. 3.5.1 (http://www.r-project.org).

### 2.5 Role of the funding source

The VIVIT research institute was supported by the Vorarlberger Landesregierung (Bregenz, Austria), which, however, exerted no influence on the present work in any way.

## 3 Results

### 3.1 Patient characteristics

Characteristics of the included subjects at the HS and at the CVOS baseline are summarized in Table 1. At the CVOS baseline, subjects were about 15 years older (median 14.6 years, IQR 11.0–19.0 years), their BMI was higher (by 3%; *p*<0.001 (Wilcoxon test)), and the prevalence of smoking lower (42%; *p*<0.001 (McNemar test)) than at the HS. In addition, comparing TC measured at the CVOS baseline (TC_CVOS;_ median=5.30 mmol/L, IQR=4.55–6.05 mmol/L) and TC measured at the HS (TC_HS_; median=6.18 mmol/L, IQR=5.40–6.99 mmol/L) revealed a decline of 14% (*p*<0.001 (Wilcoxon test)).

**Table 1:** Patient characteristics.

	HS	CVOS	p-value
	*N* = 1090	*N* = 1090	
Age (years), median (IQR)	51 (43–58)	66 (58–72)	<0.001
Male gender (%)	64.5	64.5	1
BMI (kg/m^2^), median (IQR)	26 (24–29)	27 (25–30)	<0.001
TC (mmol/l), median (IQR)	6.18 (5.40–6.99)	5.30 (4.55–6.05)	<0.001
Blood pressure, systolic (mmHg), median (IQR)	140 (125–150)	135 (125–150)	0.106
Current smoking (%)	28.6	16.5	<0.001
Statin intake (%)	0.0	45.9	<0.001

Characteristics of patients assessed at the health survey (HS) and at the baseline of the cardiovascular observation study (CVOS). The median time between HS and CVOS was 15 years. IQR denotes interquartile range. Data were obtained from single human samples and p-values were produced from paired McNemar test for categorical and Wilcoxon test for continuous variables, respectively.

### 3.2 Association between TC and the presence and extent of CAD

**Fig. 2:**
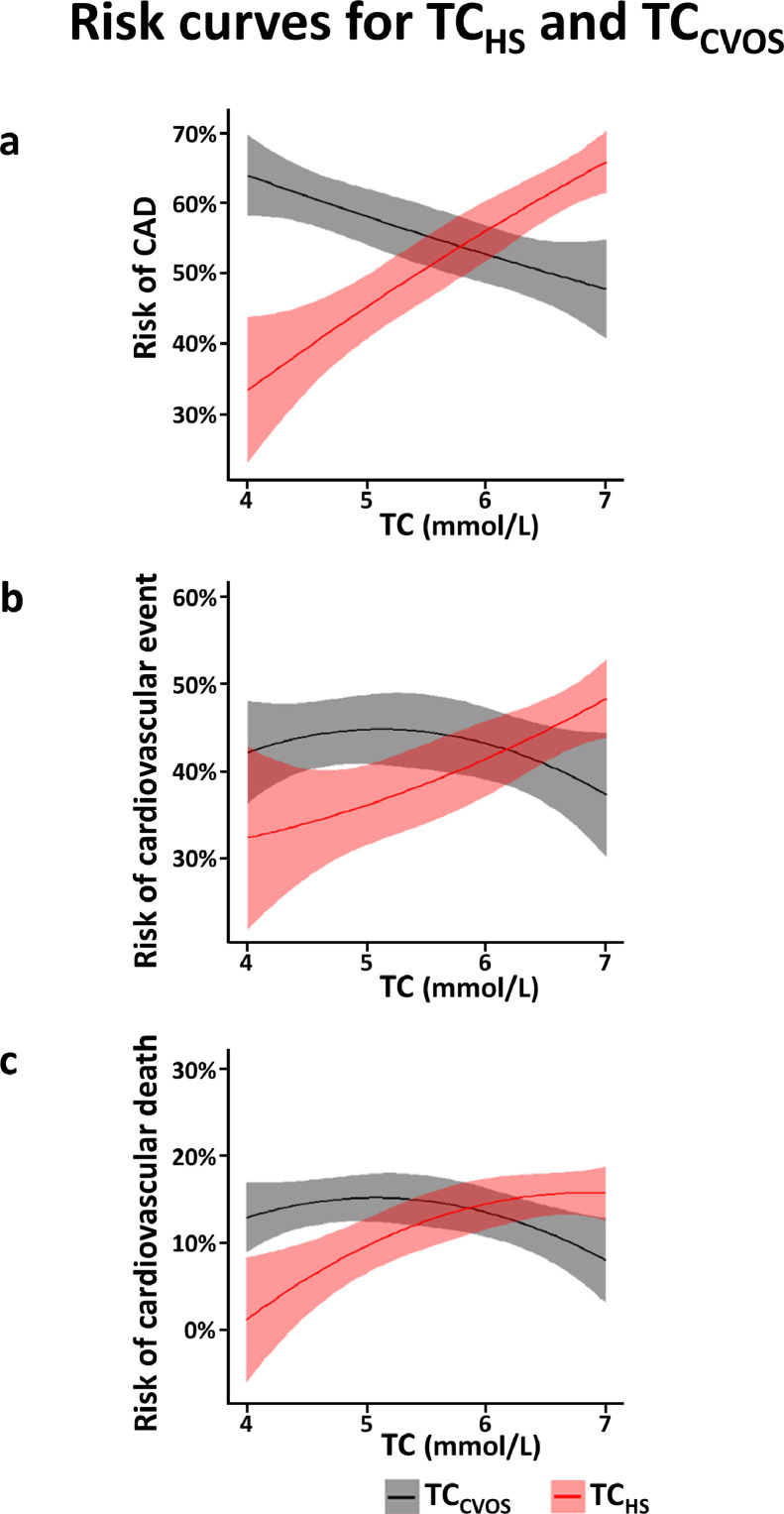
Risk curves for TC_HS_ and TC_CVOS_. The risk curves are calculated as second order polynomial fit with 95% confidence interval for the presence of CAD at CVOS baseline (a) or for suffering a cardiovascular event (b) or cardiovascular death (c) during follow up. Data were obtained from single human samples.

**Fig. 3:**
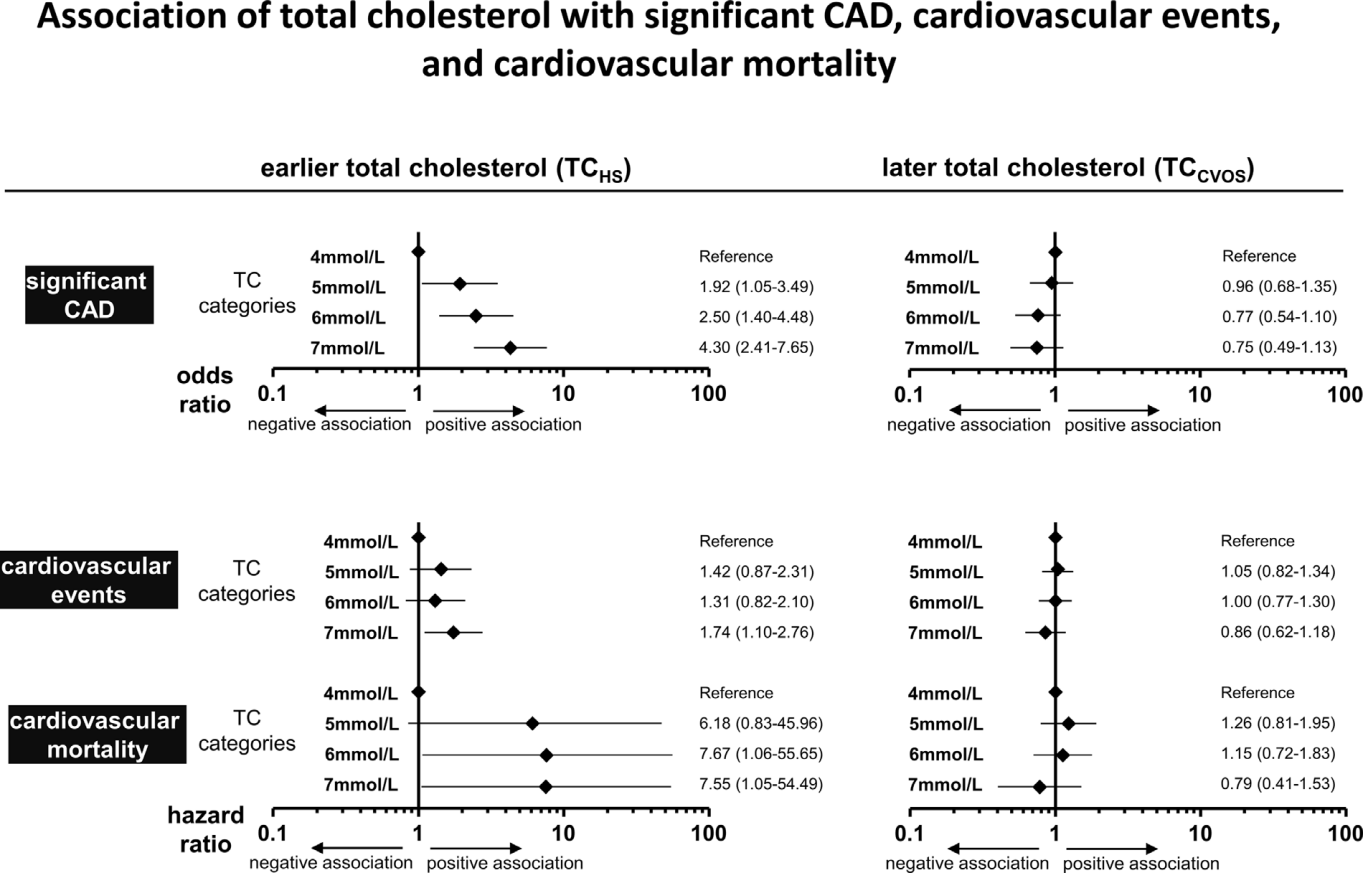
Association of total cholesterol (TC) assessed at the health survey (HS) and at the baseline of the cardiovascular observation study (CVOS) with CAD, with cardiovascular events, and with cardiovascular mortality. Forest plots represent odds ratios and hazard ratios with 95% confidence interval of binary logistic regression analyses and Cox regression analyses for the association between TC and significant CAD and between TC and cardiovascular events or cardiovascular death, respectively. Models were adjusted for age, ∆age, gender, BMI, systolic blood pressure, eGFR, smoking, and T2DM status. TC was measured either earlier in the HS or later at the baseline of the CVOS and stratified into four categories reflecting the categorization of the ESC/EAS-SCORE charts. Data were obtained from single human samples.

615 (56%) of our 1090 patients had significant CAD at angiography, the other 475 did not. To analyze the association of TC with significant CAD, OR was calculated using the lowest TC category (4 mmol/L) as reference. Fig. 2A shows that the increase in CAD prevalence matched the increase of TC_HS_ categories, but appeared to run contrary to the increase of TC_CVOS_. Results generated by logistic regression further demonstrated that only TC_HS_, but not TC_CVOS_, was significantly associated with CAD (Fig. 3A). Comparing the highest TC category to the reference category, TC_HS_ proved to be a significant predictor of CAD, even after adjustment for the CVOS baseline parameters age, ∆age, gender, BMI, systolic blood pressure, eGFR, current smoking status, and T2DM status, resulting in an adjusted OR of 4.30 [2.41–7.65]; *p*<0.001. In contrast, no significant association was found between TC_CVOS_ and significant CAD in the same model (adj. OR = 0.75 [0.49–1.13]; *p* = 0.168). This was true in women and men as well as in elderly (≥65 years) and younger subjects (**supplementary Table 1**). When TC was not used categoricaly but as a continuous variable in regression models, again TC_HS_ but not TC_CVOS_ was significantly associated with significant CAD (**supplementary Table 2**). We found no significant impact of any possible confounders, including age and gender, on the association between TC and significant CAD (**supplementary Table 3**).

Furthermore, ANCOVA revealed that TC_HS_ was also significantly associated with the extent of CAD (*F* = 25.83; *p*<0.001) and also with the severity of CAD (*F* = 37.98; *p*<0.001), applying the above-described adjustment model. In contrast, TC_CVOS_ predicted neither the extent of CAD (*F* = 1.34; *p* = 0.247) nor the severity (*F* = 0.48; *p* = 0.488) in ANCOVA. For the used adjustment models, there was no multicollinearity between continuous predictor variables (**supplementary Table 4**).

We also examined the value of TC to improve prediction of angiographically determined significant CAD. Comparing the area under the curve (AUC) of receiver operating characteristics (ROC), revealed that TC_HS_ was the single best predictor of significant CAD (AUC=0.615), surpassing TC_CVOS_, and also LDL-C_CVOS_ and high density lipoprotein-cholesterol (HDL-C_CVOS_) which have been measured at the CVOS baseline (AUC= 0.567, 0.572, and 0.595, respectively; **supplementary Table 5**). When TC_HS_ was added to a basic prediction model comprising age, ∆age, gender, BMI, systolic blood pressure, eGFR, smoking, and T2DM status, the AUC of ROC increased by 0.029 (*p* = 0.002 (DeLong's test)). In contrast, adding TC_CVOS_ instead of TC_HS_, to the respective model did not significantly improve the model`s power to predict CAD (∆AUC=0.004, *p* = 0.229 (DeLong's test), **supplementary Figure 2** and **supplementary Table 6**).

Taking into account the widely used lipid markers LDL-C and HDL-C, measured at the CVOS baseline, TC_HS_ also significantly increased the power of a prediction model, additionally comprising these lipid markers (∆AUC=0.035, *p* = 0.001 (DeLong's test), **supplementary Table 6**).

### 3.3 Association between TC and cardiovascular risk

In total, 462 patients developed a cardiovascular event during follow-up (42.4% of the study population), 377 died (34.6% of the study population), of whom 142 succumbed to cardiovascular death (13.0% of the study population). The risk increase for cardiovascular events and cardiovascular death was consistent with increasing TC_HS_, but this was not the case for TC_CVOS_ (Fig. 2**B, C**). Cox regression analysis applying adjustment as described above revelead that only TC_HS_ was significantly associated with cardiovascular events and with cardiovascular death, but not TC_CVOS._ (Fig. 3). Cardiovascular survival curves are depicted in **supplementary Figure 3**. TC_HS_ at the highest category was associated with a 1.74-fold increase [1.10–2.76] in cardiovascular events compared with the lowest category (*p* = 0.018) according to Cox regression analysis. In contrast, TC_CVOS_ was not significantly associated with cardiovascular events (HR=0.86 [0.62–1.18]; *p* = 0.345). Moreover, we observed a significantly increased risk for cardiovascular mortality in the highest vs. the lowest category of TC_HS_ (HR= 7.55 [1.05–54.49]; *p* = 0.045), but not TC_CVOS_ (HR= 0.79 [0.41–1.53]; *p* = 0.489). Similar results were obtained, when TC was used as continuous variable (**supplementary Table 2**).

500 patients have started taking statins after the HS and 590 have remained free of statins. Between HS and CVOS, TC declined with equivalent doses of statin intake (*r* = 0.39; *p*<0.001 (Spearman test)). Compared to the total study population with a decline of 14% between HS and CVOS, TC of statin-naïve subjects only declined by 7% (median TC_HS_ of statin-naive =5.9 mmol/L vs. median TC_CVOS_ of statin-naive =5.5 mmol/L; *p*<0.001 (Wilcoxon test)). Thus statin treatment was additionally included in the regression model, but this only marginally affected the significant association between TC_HS_ and (i) CAD, (ii) cardiovascular events, and (iii) cardiovascular death. Of note, the same was true if only statin-naïve patients (*n* = 590) were analyzed (**supplementary Table 2**). Risk curves for TC of statin-naïve patients are depicted in **supplementary Figure 4**.

The increase of power to predict cardiovascular events and cardiovascular mortality after incorporation of TC_HS_ into prediction models is summarized in **supplementary Table 7**. This increase was clearly attenuated and failed significance (according to DeLong's test) if TC_CVOS_ was added, instead of TC_HS_ (cardiovascular events ∆AUC=0.001, *p* = 0.847; cardiovascular mortality ∆AUC=0.004, *p* = 0.172). Moreover, TC_HS_ also increased the power of a model, which additionally comprised lipid markers LDL-C_CVOS_ and HDL-C_CVOS_, to predict cardiovascular events (∆AUC=0.015, *p* = 0.041) and cardiovascular mortality (∆AUC=0.011, *p* = 0.004).

### 3.4 Improvement of the power of the ESC/EAS-SCORE for the prediction of cardiovascular mortality

**Fig. 4:**
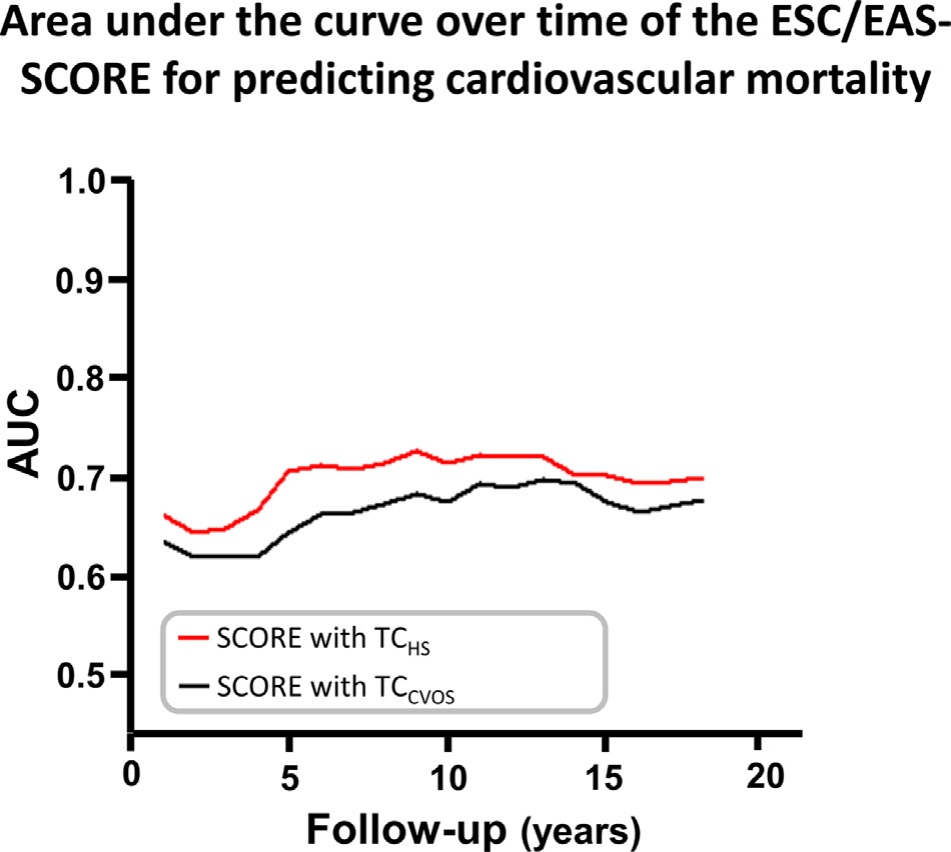
Area under the curve over time of the ESC/EAS-SCORE for predicting cardiovascular mortality. The chart indicates the area under the curve (AUC) over time based on the ESC/EAS-SCORE chart for low risk European countries predicting cardiovascular mortality. SCORE comprises TC, age, gender, the status of current smoking and systolic blood pressure. The plots represent the AUC for SCORE if containing TC measured at the baseline of the CVOS (TC_CVOS_, black line) and for SCORE if containing TC measured earlier at the HS (TC_HS_, red line). Data were obtained from single human samples.

Finally, we compared results of the ESC/EAS-SCORE comprising either TC_HS_ or TC_CVOS_ measurements. Regarding the four risk categories proposed by the 2019 ESC/EAS guidelines (ranging from low to very high for 10-year risk of cardiovascular death), 18% of patients were reclassified when using TC_HS_ instead of TC_CVOS_: 20 patients in a one-step lower risk category and 171 patients in a one-step higher risk category. Comparing the highest to the lowest risk category of the ESC/EAS-SCORE using Cox regression, the HR for the cardiovascular death was 11.18 [5.52–22.61]; *p*<0.001) when the ESC/EAS-SCORE was built with TC_HS_ and 7.29 [3.80–13.95]; *p*<0.001) when the ESC/EAS-SCORE was built with TC_CVOS_. The predictive power of the ESC/EAS-SCORE was significantly higher when the earlier TC_HS_ instead of the later TC_CVOS_ measurements were used, as evaluated by the integrated discrimination improvement (IDI) index (IDI=0.017, *p* = 0.005) and the continuous net reclassification improvement (cNRI) index (cNRI=0.301, *p*<0.001). Accordingly, the AUC of ESC/EAS-SCORE using TC_HS_ instead of TC_CVOS_ was higher during follow-up time (Fig. 4).

## 4 Discussion

This prospective study was performed in an Austrian cohort of patients who underwent coronary angiography and who had also participated in a HS 15 years earlier. TC measured earlier in life, at the HS, was a significant predictor of coronary atherosclerosis at angiography, of cardiovascular events, and of cardiovascular mortality during the follow-up of the CVOS. Compared to the TC measurement at the CVOS baseline, TC measured 15-years earlier was significantly more valuable in that it improved the prediction of cardiovascular risk. Moreover, earlier TC significantly improved the cardiovascular risk prediction over and above LDL-C and HDL-C concentrations as measured at the CVOS baseline. We also found that, when earlier TC readings were used for the calculation of the ESC/EAS-SCORE, 18% of patients are reclassified regarding risk categories and that the prediction of cardiovascular mortality was more accurate compared to the use of later TC readings. Our results thus indicate that TC assessed earlier in life, when people are healthy and untreated, are worth being recorded to improve risk prediction much later in life.

To the best of our knowledge, the present study is the first investigation comparing the power of two TC measurements separated by a median time span of 15 years, for predicting the presence of coronary atherosclerosis and the subsequent incidence of cardiovascular events over another time span of up to 19 years. Thus, the study is the first of its kind, covering a period of up to 33 years.

We found that TC declines with age and found that midlife TC justifies the classical understanding of TC in cardiovascular risk prediction, whereas TC 15 years later in the same patients does not. In line with our data, previous large studies have found that decline of TC concurs with advancing age [25], [26], [27], [28]. Although TC declines also in subjects without lipid lowering medication [26], statin treatment is a driving force for this decline and has been suggested to range from modest [25] to important [27,28]. Moreover, TC of our elderly patients (assessed at CVOS) was not associated with their cardiovascular risk. This finding is similar to previous observational studies reporting that the causal relation between cholesterol (TC and LDL-C) and cardiovascular disease was absent or even inverse at old age [29], [30], [31]. Hence the real risk may be underestimated [32]. As demonstrated in the SATURN [33] and MIRACL [6] trials, neither LDL-C nor TC of patients on statin treatment were predictive of cardiovascular events [6,33]. Nevertheless, in our study, when taking statin treatment into account, TC measured earlier in life (mid adulthood) was still superior to TC measured later in life when it comes to cardiovascular risk prediction, even in statin-naïve patients. This indicates the presence of further, statin-independent effects compromising the predictive power of TC in older subjects. For example, the strength of association between the risk of ischaemic heart disease and cholesterol has been reported to decrease with age [34]. However, the nature of these effects along with the impact e.g. of comorbidities [29], drug intake, or drug interactions in elderly [35] remains vague and calls for further research.

Risk prediction is perceived to be most relevant for older patients, as, according to the ESC/EAS SCORE charts, most risk factors misleadingly appear to have no impact on people in their 40ies. They have a very low (10 year fatal) risk profile irrespective of any parameter assessed by SCORE, including TC.

Nevertheless, the present study findings clearly recommend that TC reading should be done at midlife or even earlier and saved for later in life when cardiovascular risk prediction becomes a bigger issue.

Therefore our results confirm previous studies on the association of the life-long burden of high TC levels with the lifetime risk of cardiovascular disease [32,36], and advocate an early start of screening and treatment [37,38]. In line with our findings and conclusions, a recent meta-analysis comprising 38 cohorts demonstrated that inclusion of repeated risk factor measurements, in particular of TC, into risk prediction models improves the accuracy of a 5-year cardiovascular disease prediction [39]. Similarly, insights from the Framingham study demonstrated that TC trajectories, which comprised TC measurements over a 35 year period, improved prediction of incident atherosclerotic cardiovascular disease more than the use of a single TC measurement [40]. Moreover, a recent study from Germany found that SCORE estimates using 10 year old TC readings were suited to identify patients at high cardiovascular disease risk with high sensitivity and specificity [41]. However, this population-based study, in contrast to our investigation on angiographied coronary patients, found only minor changes of earlier versus later TC over time (5.88 to 5.73 mmol/*l* = 3% decrease), and, thus, no difference regarding prediction of cardiovascular disease. Consequently, one might reason that the decline of TC between midlife and advanced age is not necessarily linked to a better outcome and that current readings of patients at an advanced age are unsuited for cardiovascular risk prediction.

Clinical routine is usually based on current measurements. Although this is ideal for most health issues, the value of older data is often underestimated. The concept of lifelong exposure to atherogenic lipoproteins is indisputable [32,36]. Similar to genetic analyses assessing the lifetime risk [32], early measurement enables early diagnosis and therapy. The present study data clearly corroborate the shift in recent guidelines towards earlier and more aggressive statin treatment [42]. To achieve maximum benefit, TC measurements should not be limited to patients with evident high cardiovascular risk but be conducted routinely, especially in young and healthy subjects and such readings should be well documented. Clearly, our study adds evidence to that important notion. In this context, the belief that at advanced age risk factors other than TC are more important, should be put into perspective. In view of our data, this only applies to current TC readings in older patients, but not to past readings assessed at midlife (as in our study) or the genetic predisposition for high cholesterol levels [32].

This study has strengths and limitations. Particular strengths include the design of the study. The study was done in a well-defined geographical area with low migration. It comprised data from middle-aged statin-naïve participants and of the same participants a median of 15 years later when they had become patients referred to coronary angiography for the evaluation of stable CAD, reflecting a real world situation. Further strengths of the study are the extremely high follow-up rate of >99% and the fact that all samples of the mentioned two measurements (at HS and at CVOS) were analyzed in the same laboratories. To differentiate the nature of CAD, we assessed its extent and severity. A more detailed view on the concept of CAD has been given previously [43]. A potential limitation is that our study participants were, at least at the time point of HS recruitment, healthy volunteers and might represent a particularly health-conscious Caucasian population. Hence, we cannot claim for certain that our results apply to the general populations or to other ethnicities. Furthermore, we compared TC of patients only at two time points, at a mean age of 51 and 66 years. More time points, including TC measurement in even younger subjects, would be necessary to determine the optimal timing of TC measurement. In addition, only data from TC measurements, and not on lipoprotein lipids, were available from the HS. Finally, about half of our patients, especially those with high TC at HS have started taking statins at any time point after HS recruitment. Though we have no data about their adherence to medical treatment [44], this of course impacted TC concentrations measured at CVOS. On the other hand, this reflects clinical reality and underlines the value and importance of data assessed earlier in life and prior to lipid lowering therapy. That said, comparable results were obtained when we limited the analysis to statin-naïve patients. Of note, this should not lead to any misleading conclusions about the effect of statins to modify CV risk.

Routine measurements of TC starting early in life with young and healthy subjects are of dual benefit: such early readings, as shown here for mid adulthood subjects, are more valuable for cardiovascular risk prediction than readings obtained later in life. What's more, they enable earlier treatment of patients at risk.

Although present scores, including the ESC/EAS-SCORE, are using TC, applying the same study setting to measurements of other lipids such as LDL-C, apolipoprotein B or also ceramides of course would be of great interest. Apart from the present study enrolling angiographied coronary patients in Austria, and given the fact that cohort data are missing for most countries [12], investigations on the power of TC measured earlier in life to predict significant CAD as well as cardiovascular events in other cohorts and populations are warranted. Finally, after having compared midlife to elderly TC readings here, a comparison between young and midlife TC for risk prediction in later life may be insightful as well.

In conclusion, our study shows that when comparing earlier vs. later TC readings, the former is the more valuable predictor of cardiovascular risk in elderly patients.

## Contributors

A.L. researched data and wrote the manuscript. H.U. researched data and contributed to discussion and reviewed/edited the manuscript. A.M. researched data and reviewed/edited the manuscript. C.H.S. researched data, designed and managed the project, and reviewed the manuscript. A.V. researched data and managed the project. P.F., B.F., E.M.B., W.B., G.N., and E.Z. contributed to discussion and reviewed the manuscript. H.D. and H.C designed and managed the project, contributed to discussion, reviewed the manuscript, and are the guarantors of this work and, as such, had full access to all the data in the study and take responsibility for the integrity of the data and the accuracy of the data analysis.

## Data sharing statement

The data that support the findings of this study are available from the corresponding author upon reasonable request.

## Declaration of Competing Interest

A.L. has nothing to disclose, H.U. has nothing to disclose, A.M. has nothing to disclose, C.H.S. has nothing to disclose, A.V. has nothing to disclose, P.F. has nothing to disclose, B.F. has nothing to disclose, E.M.B. has nothing to disclose, W.B. has nothing to disclose, G.N. has nothing to disclose, E.Z. has nothing to disclose, H.D. has nothing to disclose, and H.C. has nothing to disclose.

## Appendix Supplementary materials

Image, application 1

Image, application 1
