# Supplementary material for: Value of total cholesterol readings earlier versus later in life to predict cardiovascular risk
Source: eBioMedicine. 2021 May 14;67:103371. doi: 10.1016/j.ebiom.2021.103371 (PMC8138461; doi:10.1016/j.ebiom.2021.103371)
Supplement: Supplementary file 1 [file mmc1.docx]

**Supplementary data**

**
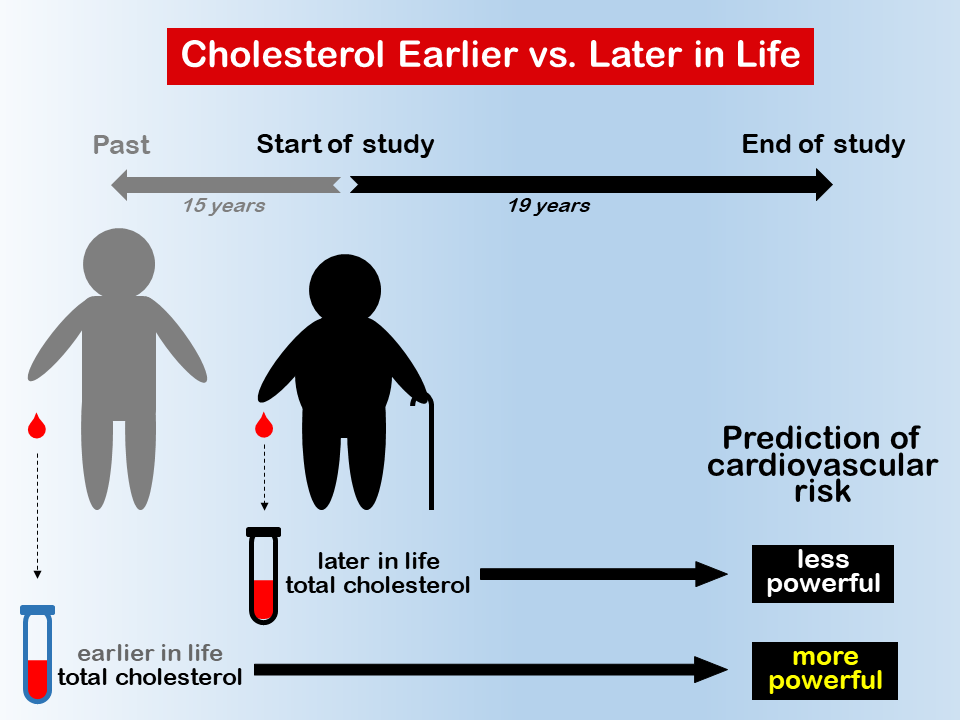
**

**Supplementary Figure 1: Study summary: Cholesterol earlier versus later in life.** The illustration summarizes the conclusion in that it demonstrates that total cholesterol concentration assessed earlier in life during mid adulthood is more valuable for predicting cardiovascular risk than measurements later in life.

***
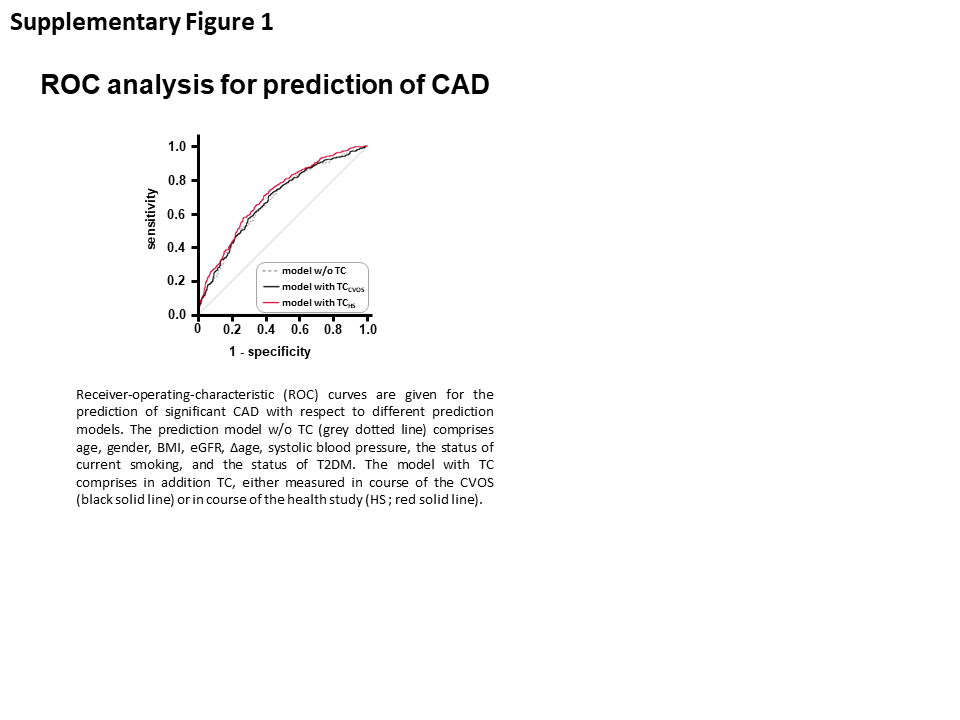
***

**Supplementary Figure 2: Receiver-operating-characteristic (ROC) analysis for the prediction of coronary artery disease (CAD).** ROC curves are given for the prediction of significant CAD with respect to different prediction models. The prediction model without (w/o) TC (grey dotted line) comprises age, gender, BMI, eGFR, ∆age, systolic blood pressure, the status of current smoking, and the status of T2DM. The model with TC comprises TC in addition to the aforementioned parameters, either measured at the baseline of the cardiovascular observation study (CVOS; black solid line) or at the health survey (HS; red solid line). Data were obtained from single human samples.


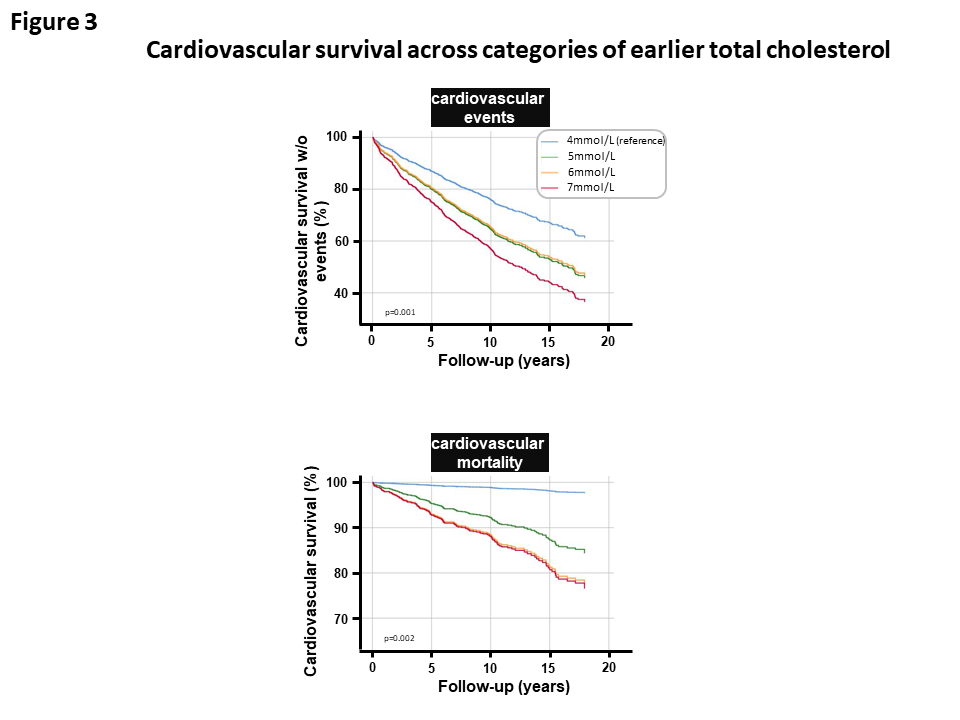


**Supplementary Figure 3: Cardiovascular survival across categories of TC_HS_.** The survival curves indicate survival without cardiovascular event or cardiovascular survival according to TC categories, measured in the HS. Data were obtained from single human samples. The p-value was produced from Cox proportional hazards regression analysis and is given for trend over categories.

*
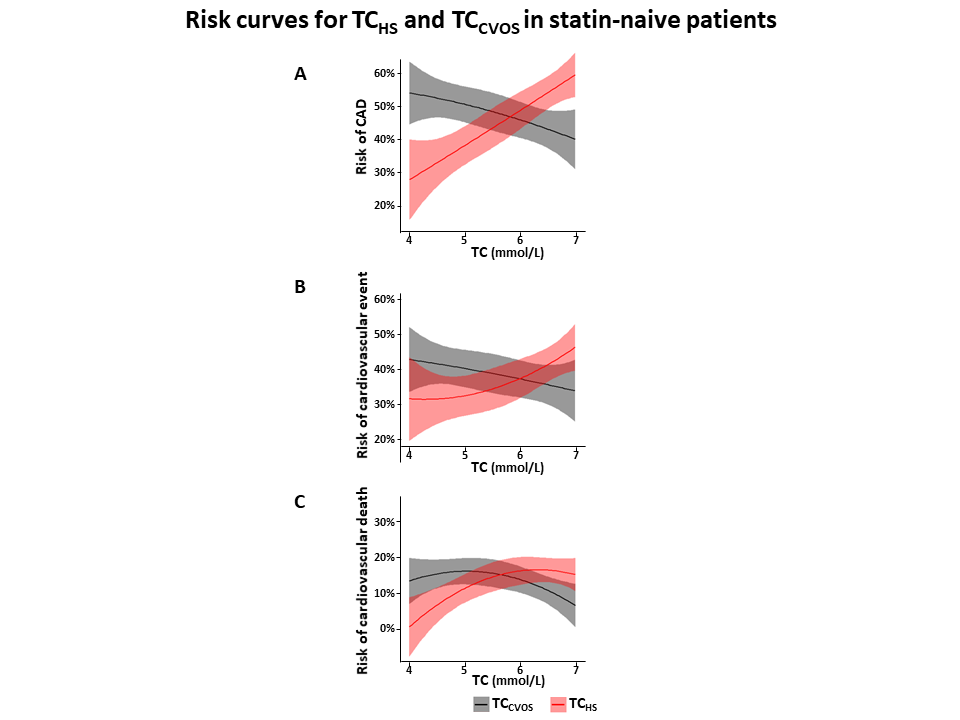
*

**Supplementary Figure 4: Risk curves for TC_HS_ and TC_CVOS_ in statin-naive patients.** The risk curves are calculated for the presence of CAD at CVOS baseline (A) or for suffering a cardiovascular event (B) or cardiovascular death (C) during follow up. Only statin-naive patients (n=590) were included. Data were obtained from single human samples.

| **subgroups** | **n** | **TC measured**  **at** | **TC categories** | **OR with [95%CI]** |
| --- | --- | --- | --- | --- |
| **all** | 1090 | HS | 4  5  6  7 | Reference  1.92 [1.05-3.49]  2.50 [1.40-4.48]  4.30 [2.41-7.65] |
|  |  | CVOS | 4  5  6  7 | Reference  0.96 [0.68-1.35]  0.77 [0.54-1.10]  0.75 [0.49-1.13] |
| **women** | 387 | HS | 4  5  6  7 | Reference  3.14 [0.96-10.28]  3.96 [1.27-12.36]  7.74 [2.48-24.14] |
|  |  | CVOS | 4  5  6  7 | Reference  1.25 [0.67-2.34]  0.76 [0.40-1.45]  1.04 [0.51-2.13] |
| **men** | 703 | HS | 4  5  6  7 | Reference  1.61 [0.78-3.32]  2.14 [1.05-4.36]  3.53 [1.75-7.10] |
|  |  | CVOS | 4  5  6  7 | Reference  0.85 [0.56-1.29]  0.78 [0.50-1.22]  0.66 [0.39-1.13] |
| **older**  (≥65years) | 575 | HS | 4  5  6  7 | Reference  3.34 [1.21-9.21]  4.21 [1.60-11.07]  6.77 [2.58-17.76] |
|  |  | CVOS | 4  5  6  7 | Reference  0.75 [0.46-1.22]  0.67 [0.42-1.14]  0.83 [0.45-1.53] |
| **younger**  (<65years) | 515 | HS | 4  5  6  7 | Reference  1.40 [0.66-2.96]  1.81 [0.86-3.82]  3.48 [1.67-7.24] |
|  |  | CVOS | 4  5  6  7 | Reference  1.30 [0.79-2.13]  0.94 [0.55-1.59]  0.73 [0.41-1.33] |

**Supplementary Table 1. Association of total cholesterol (TC) assessed at the health survey (HS) and at the baseline of the cardiovascular observation study (CVOS) with presence of significant CAD in patient subgroups.**  Data represent odds ratio (OR) with 95% confidence interval (CI) of binary logistic regression analyses for the association between TC and significant CAD. TC was measured either earlier in the HS (median TC_HS_=6.2mmol/l) or later at the baseline of the CVOS (median TC_CVOS_=5.3mmol/l) and stratified into four categories reflecting the categorization of the ESC/EAS-SCORE charts. Models were adjusted for age, ∆age, gender, BMI, systolic blood pressure, eGFR, smoking, and T2DM status. For subgroup analyses, patients were stratified according to gender (female/male) and age (≥65 years/<65years). Data were obtained from single human samples.

| **Outcome** | **TC measured**  **at** | **model** | **patients included** | **OR / HR with [95%CI]** | **p-value** |
| --- | --- | --- | --- | --- | --- |
| Presence of sig. CAD | HS | 1  2  3  1* | all (n=1090)  all (n=1090)  all (n=1090)  statin-naïve only (n=590) | 1.53 [1.33-1.77]  1.42 [1.23-1.64]  1.40 [1.22-1.62]  1.51 [1.25-1.82] | <0.001  <0.001  <0.001  <0.001 |
| Presence of sig. CAD | CVOS | 1  2  3  1* | all (n=1090)  all (n=1090)  all (n=1090)  statin-naïve only (n=590) | 0.88 [0.77-1.01]  0.95 [0.83-1.09]  0.99 [0.86-1.14]  0.92 [0.77-1.11] | 0.058  0.478  0.897  0.404 |
| Cardiovascular event | HS | 1  2  3  1* | all (n=1090)  all (n=1090)  all (n=1090)  statin-naïve only (n=590) | 1.17 [1.07-1.28]  1.14 [1.04-1.26]  1.13 [1.03-1.25]  1.21 [1.06-1.38] | 0.001  0.007  0.011  0.006 |
| Cardiovascular event | CVOS | 1  2  3  1* | all (n=1090)  all (n=1090)  all (n=1090)  statin-naïve only (n=590) | 0.94 [0.86-1.04]  0.97 [0.88-1.07]  0.99 [0.90-1.09]  0.87 [0.75-1.00] | 0.224  0.577  0.786  0.050 |
| Cardiovascular death | HS | 1  2  3  1* | all (n=1090)  all (n=1090)  all (n=1090)  statin-naïve only (n=590) | 1.23 [1.02-1.41]  1.24 [1.03-1.49]  1.23 [1.02-1.48]  1.29 [1.01-1.65] | 0.030  0.024  0.029  0.042 |
| Cardiovascular death | CVOS | 1  2  3  1* | all (n=1090)  all (n=1090)  all (n=1090)  statin-naïve only (n=590) | 0.93 [0.78-1.11]  0.92 [0.77-1.11]  0.93 [0.78-1.12]  0.85 [0.66-1.09] | 0.436  0.378  0.452  0.200 |

**Supplementary Table 2. Association of TC with the presence of CAD, cardiovascular events, and cardiovascular mortality.** TC was measured either earlier, in the course of the HS (median TC_HS_=6.2mmol/l), or later, in the course of the CVOS (median TC_CVOS_=5.3mmol/l). Odds ratios (OR) and hazard ratios (HR), respectively, were calculated for TC used as continuous variable, applying adjustment for age, ∆age (if applicable), gender, BMI, systolic blood pressure, eGFR, the current smoking status, and the T2DM status (model 1). Model 2 comprises all variables of model 1 and in addition the status of statin treatment. Model 3 comprises all variables of model 1 and in addition statin doses equivalents. Model 1* comprises all variables as mentioned in model 1 but analyzes only statin-free patients (n= 590; median TC_HS_=5.9mmol/l, median TC_CVOS_=5.5mmol/l). P-values were produced from logistic regression and Cox proportional hazards regression analysis, respectively. Data were obtained from single human samples.

| **outcome** | **independent variable** | **moderator** | **p-value of interaction term** |
| --- | --- | --- | --- |
| sig. CAD | TC_HS_ | age  gender  BMI  BP sys  eGFR  Smoking  Diabetes  Δage | 0.702  0.247  0.146  0.378  0.058  0.856  0.341  0.866 |

**Supplementary Table 3. Moderator analysis of the association between TC_HS_ and significant CAD with parameters included in adjustment model as moderators.**

Moderators age, ∆age, gender, BMI, systolic blood pressure (BP sys), eGFR, smoking, and type 2 diabetes mellitus (T2DM) status were included in a binary regression model with the outcome sig. CAD and the independent predictor TC_HS_. P-values are given for the interaction of the independent variable and each moderator in the logistic regression model. Data were obtained from single human samples.

| \| **Variables** \| **Model 1** \| \| **Model 2** \| \| **Model 3** \| \| **Model 4** \| \| \| --- \| --- \| --- \| --- \| --- \| --- \| --- \| --- \| --- \| \|  \| **tolerance** \| **VIF** \| **tolerance** \| **VIF** \| **tolerance** \| **VIF** \| **tolerance** \| **VIF** \| \| **age_CVOS_** \| **0.75** \| **1.34** \| **0.72** \| **1.39** \| **0.77** \| **1.29** \| **0.74** \| **1.36** \| \| **BMI_CVOS_** \| **0.91** \| **1.10** \| **0.85** \| **1.17** \| **0.91** \| **1.10** \| **0.85** \| **1.17** \| \| **sys BP _CVOS_** \| **0.93** \| **1.08** \| **0.92** \| **1.08** \| **0.93** \| **1.08** \| **0.92** \| **1.08** \| \| **LDL-C_CVOS_** \| **n.a.** \| **n.a.** \| **0.88** \| **1.14** \| **n.a.** \| **n.a.** \| **0.20** \| **5.13** \| \| **HDL-C_CVOS_** \| **n.a.** \| **n.a.** \| **0.72** \| **1.39** \| **n.a.** \| **n.a.** \| **0.69** \| **1.46** \| \| **eGFR_CVOS_** \| **0.86** \| **1.16** \| **0.84** \| **1.20** \| **0.87** \| **1.15** \| **0.83** \| **1.21** \| \| **∆age** \| **0.95** \| **1.05** \| **0.95** \| **1.05** \| **n.a.** \| **n.a.** \| **n.a.** \| **n.a.** \| \| **TC_HS_** \| **0.97** \| **1.03** \| **0.92** \| **1.09** \| **n.a.** \| **n.a.** \| **n.a.** \| **n.a.** \| \| **TC_CVOS_** \| **n.a.** \| **n.a.** \| **n.a.** \| **n.a.** \| **0.95** \| **1.05** \| **0.19** \| **5.31** \| \| **gender** \| **0.94** \| **1.06** \| **0.82** \| **1.23** \| **0.93** \| **1.08** \| **0.81** \| **1.23** \| \| **T2DM** \| **0.93** \| **1.07** \| **0.88** \| **1.13** \| **0.91** \| **1.09** \| **0.88** \| **1.14** \| \| **smoking** \| **0.90** \| **1.12** \| **0.89** \| **1.12** \| **0.90** \| **1.12** \| **0.89** \| **1.13** \| |
| --- | --- | --- | --- | --- | --- | --- | --- | --- | --- | --- | --- | --- | --- | --- | --- | --- | --- | --- | --- | --- | --- | --- | --- | --- | --- | --- | --- | --- | --- | --- | --- | --- | --- | --- | --- | --- | --- | --- | --- | --- | --- | --- | --- | --- | --- | --- | --- | --- | --- | --- | --- | --- | --- | --- | --- | --- | --- | --- | --- | --- | --- | --- | --- | --- | --- | --- | --- | --- | --- | --- | --- | --- | --- | --- | --- | --- | --- | --- | --- | --- | --- | --- | --- | --- | --- | --- | --- | --- | --- | --- | --- | --- | --- | --- | --- | --- | --- | --- | --- | --- | --- | --- | --- | --- | --- | --- | --- | --- | --- | --- | --- | --- | --- | --- | --- | --- | --- | --- | --- | --- | --- | --- | --- | --- | --- | --- |

**Supplementary Table 4: Collinearity statistics.** Data of collinearity statistics are given for predictor variables as tolerance and variance inflation factor (VIF) in a linear regression model for the prediction of extent of significant CAD. The regression model comprises variables age, gender, BMI, eGFR, systolic blood pressure (sys BP), diabetes and current smoking status, ∆age, and TC (model 1 &3) or in addition LDL-C and HDL-C (model 2&4). VIF for all covariates was clearly below 5 in models 1-3 (model 4 was not applied). Data were obtained from single human samples.

| \| **Predictor** \| **AUC from ROC to predict CAD** \| \| --- \| --- \| \| **TC_HS_** \| **0.615** \| \| **HDL-C_CVOS_** \| **0.595** \| \| **LDL-C_CVOS_** \| **0.572** \| \| **TC_CVOS_** \| **0.567** \| \| **Age_CVOS_** \| **0.544** \| \| **BMI_CVOS_** \| **0.527** \| \| **sys BP_CVOS_** \| **0.522** \| \| **eGFR_CVOS_** \| **0.508** \| |
| --- | --- | --- | --- | --- | --- | --- | --- | --- | --- | --- | --- | --- | --- | --- | --- | --- | --- | --- |

**Supplementary Table 5. ROC analysis.** The table summarizes area under the curve (AUC) for receiver-operating-characteristic (ROC) curves of single continuous predictors for the prevalence of significant CAD. AUCs are limited to the range between 0.5 – 1.0 for better comparison by reversing predictors if applicable. Data were obtained from single human samples.

| **Models predicting sig. CAD** | **AUC** | **OMQ** | **z** | **p-value** |
| --- | --- | --- | --- | --- |
| basic model (w/o TC)^1^ | 0.676 | 0.64 | - | - |
| basic model with TC_HS_ ^2^ | 0.705 | 0.67 | 3.024 ^2 vs. 1^ | 0.002 ^2 vs. 1^ |
| basic model with TC_CVOS_ ^3^ | 0.680 | 0.65 | 1.204 ^3 vs. 1^ | 0.229 ^3 vs. 1^ |
| lipid model (w/o TC) ^4^ | 0.689 | 0.66 | - | - |
| lipid model with TC_HS_^5^ | 0.725 | 0.69 | 3.470 ^5 vs.4^ | 0.001 ^5 vs. 4^ |

**Supplementary Table 6. C-statistics for prediction models on CAD.** The table summarizes AUC, for ROC curves and the overall model quality (OMQ) of different prediction models. The increase of AUC between two models is given by DeLong`s z-value and the respective p-value. Variables included in the basic model were age, gender, BMI, eGFR, systolic blood pressure, the current smoking status, the T2DM status, and ∆age, which were assessed at baseline of the CVOS. LDL-C and HDL-C were also assessed at baseline of the CVOS and were additionally included in the lipid model (age, gender, BMI, eGFR, systolic blood pressure, the current smoking status, the T2DM status, ∆age, LDL-C, HDL-C). TC was either from the HS or from the CVOS. Data were obtained from single human samples.

| **Models predicting cardiovascular events** | **AUC** | **OMQ** | **z** | **p-value** | **Harrell’s**  **C** | **Somers’**  **D** |
| --- | --- | --- | --- | --- | --- | --- |
| basic model (w/o TC) ^1^ | 0.638 | 0.61 | - | - | 0.616 | 0.232 |
| basic model with TC_HS_ ^2^ | 0.653 | 0.62 | 2.165 ^2 vs. 1^ | 0.030^2 vs. 1^ | 0.627 | 0.254 |
| basic model with TC_CVOS_ ^3^ | 0.639 | 0.61 | 0.193 ^3 vs. 1^ | 0.847^3 vs. 1^ | 0.616 | 0.232 |
| lipid model (w/o TC) ^4^ | 0.647 | 0.61 | - | - | 0.627 | 0.253 |
| lipid model with TC_HS_ ^5^ | 0.662 | 0.63 | 2.047^5 vs. 4^ | 0.041 ^5 vs.4^ | 0.638 | 0.276 |
| **Models predicting cardiovascular death** |  |  |  |  |  |  |
| basic model (w/o TC) ^1^ | 0.778 | 0.74 | - | - | 0.795 | 0.590 |
| basic model with TC_HS_ ^2^ | 0.785 | 0.75 | 1.614 ^2 vs. 1^ | 0.106^2 vs. 1^ | 0.799 | 0.598 |
| basic model with TC_CVOS_ ^3^ | 0.782 | 0.74 | 1.367 ^3 vs. 1^ | 0.172 ^3 vs. 1^ | 0.796 | 0.591 |
| lipid model (w/o TC) ^4^ | 0.790 | 0.75 | - | - | 0.807 | 0.613 |
| lipid model with TC_HS_ ^5^ | 0.801 | 0.76 | 2.890 ^5 vs. 4^ | 0.004 ^5 vs. 4^ | 0.813 | 0.627 |

**Supplementary Table 7. C-statistics for prediction models on cardiovascular events and cardiovascular mortality.** The table summarizes AUC (not considering time dependency), the overall model quality (OMQ), Harrell´s C and Somers´ D (considering time dependency) of different prediction models (described in supplementary Table 4) in the presence or absence of TC (assessed at HS or CVOS). P-values were produced applying DeLong's test. Data were obtained from single human samples.
