# Supplementary material for: Value of total cholesterol readings earlier versus later in life to predict cardiovascular risk
Source: eBioMedicine. 2021 May 14;67:103371. doi: 10.1016/j.ebiom.2021.103371 (PMC8138461; doi:10.1016/j.ebiom.2021.103371)
Supplement: Supplementary file 2 [file mmc2.docx]

**Caption for supplementary material**

There is one file named “Supplementary_Ebio_5” which contains 4 figures and 7 tables.

This file may be named “Supplementary figures and tables” in the final version to be published.
